# Fatigue predicts quality of life after leucine‐rich glioma‐inactivated 1‐antibody encephalitis

**DOI:** 10.1002/acn3.52006

**Published:** 2024-02-01

**Authors:** Sophie N. M. Binks, Michele Veldsman, Adam E. Handel, Saiju Jacob, Paul Maddison, Jan Coebergh, Sophia Michael, Sudarshini Ramanathan, Ava Easton, Mette Scheller Nissen, Maria Isabel Leite, David Okai, Morten Blaabjerg, Masud Husain, Sarosh R. Irani

**Affiliations:** ^1^ Oxford Autoimmune Neurology Group, Nuffield Department of Clinical Neurosciences Oxford UK; ^2^ Department of Neurology Oxford University Hospitals NHS Foundation Trust Oxford UK; ^3^ Department of Experimental Psychology University of Oxford Oxford UK; ^4^ Department of Neurology, Queen Elizabeth Hospital University Hospitals Birmingham NHS Foundation Trust Birmingham UK; ^5^ Department of Neurology, Queen's Medical Centre Nottingham University Hospitals NHS Trust Nottingham UK; ^6^ St Peter's Hospital Ashford and St Peter's NHS Hospitals Foundation Trust Chertsey UK; ^7^ Translational Neuroimmunology Group, Faculty of Medicine and Health University of Sydney Sydney New South Wales Australia; ^8^ Department of Neurology Concord Hospital Sydney New South Wales Australia; ^9^ The Encephalitis Society 32 Castlegate, Malton North Yorkshire YO17 7DT UK; ^10^ Department of Clinical Infection, Microbiology and Immunology University of Liverpool Liverpool UK; ^11^ Department of Neurology Odense University Hospital Odense Denmark; ^12^ Department of Clinical Research University of Southern Denmark Odense DK‐5000 Denmark; ^13^ Neuropsychiatry Department Maudsley Outpatients, Maudsley Hospital Denmark Hill London SE5 8AZ UK; ^14^ Nuffield Department of Clinical Neurosciences Oxford UK; ^15^ Departments of Neurology and Neurosciences Mayo Clinic Jacksonville Florida USA

## Abstract

Patient‐reported quality‐of‐life (QoL) and carer impacts are not reported after leucine‐rich glioma‐inactivated 1‐antibody encephalitis (LGI1‐Ab‐E). From 60 patients, 85% (51 out of 60) showed one abnormal score across QoL assessments and 11 multimodal validated questionnaires. Compared to the premorbid state, QoL significantly deteriorated (*p* < 0.001) and, at a median of 41 months, fatigue was its most important predictor (*p* = 0.025). In total, 51% (26 out of 51) of carers reported significant burden. An abbreviated five‐item battery explained most variance in QoL. Wide‐ranging impacts post‐LGI1‐Ab‐E include decreased QoL and high caregiver strain. We identify a rapid method to capture QoL in routine clinic or clinical trial settings.

## Introduction

Leucine‐rich glioma‐inactivated 1‐antibody encephalitis (LGI1‐Ab‐E) is the commonest form of autoimmune encephalitis (AE) in adults and typically causes seizures, behavioural and cognitive impairment.^1^, ^2^ Early immunotherapy often effectively treats seizures and can halt cognitive decline.^3^ However, longer‐term LGI1‐Ab‐E follow‐up studies highlight a potentially severe spectrum of neuropsychological impacts.^4^, ^5^ Little is known about which of these impact on quality‐of‐life (QoL).

Fatigue has emerged as a notable sequel to AE.^6^, ^7^ We previously reported fatigue as the predominant long‐term impairment in LGI1‐Ab‐E, and found it correlated with depression, anxiety, cognition and the modified Rankin Scale (mRS).^4^ Although frequently used in AE, the mRS was developed for stroke and is widely considered inadequate as an AE outcome. Recently, the Clinical Assessment Scale in Autoimmune Encephalitis (CASE) has been proposed as a more AE‐tailored scale, especially in N‐methyl‐D‐aspartate receptor‐antibody encephalitis (NMDAR‐Ab‐E).^8^ However, AE studies to date have focused on clinician‐rated outcomes, not patient‐ or carer‐ rated measures.

Here, we characterise patient‐reported QoL and evaluate carer impacts. Further, we cross‐correlate QoL with an extended battery of neuropsychiatric tools and devise an abbreviated QoL‐centred battery for use in clinical and trial settings.

## Methods

### Clinical assessment

Recruitment of 60 LGI1‐Ab‐E patients, their clinical characteristics and performance across standardised questionnaires assessing cognition, affect, fatigue, and clinician‐rated disability, was previously described (Tables S1 and S2).^4^ Here, we extend these assessments to include:

- Pathological Laughter and Crying Scale^9^: a clinician‐rated measure to assess emotionality, as we had observed LGI1‐Ab‐E patients with tearfulness to usually innocuous stimuli;
- The Neuropsychiatric Inventory Questionnaire (NPIQ)^10^: a carer‐rated measure of the patient's symptom severity (NPIQ‐S) and associated carer distress (NPIQ‐D);
- QoL. Patients rated their current and pre‐illness QoL using:
  - EQ5D5L^11^. EQ5D5L is validated across multiple diseases using two components: a five‐point index scale covering mobility, self‐care, activities of daily living, pain/discomfort and anxiety/depression and a visual analogue scale (EQ5D5L‐VAS; 0%–100%) to record health status;
  - Life Satisfaction Questionnaire (LSQ).^12^ Nine QoL domains are rated (Q1‐9): life as a whole (measuring overall QoL) in addition to self‐care, leisure, vocation, finances, sexual life, partnership relation, family life, and friends and acquaintances; each from 1 (very dissatisfying) to 6 (very satisfying).

Data were collected from 60 patients, with the exception of fatigue scales, completed by 31. Numbers of completed assessments are shown as denominators throughout. There were no significant differences between groups with and without fatigue questionnaires, other than a shorter time of illness duration (37.7 vs. 75.4 months; t(51.74) = 3.270, *p* = 0.002) (Table S3); as previously reported.^4^ Using age‐appropriate thresholds, cut‐offs for impairment were derived from manuals, published cohorts, or if no specified cut‐off available, set at two standard deviations from the healthy control value.

### Statistical methods

Detailed statistical methods are described in supplementary methods. Pre‐ and post‐acute illness scores for QoL measures (EQ5D5L‐VAS and LSQ question 1) were compared using t‐ and Wilcoxon signed‐rank tests in JASP (2019; Version 0.10.2 or R(v4.0.3)). Correlations (Spearman method) and clustering were performed and plotted in R with hmisc (v5.1.1), corrplot (v0.92) and ggpubr (v0.6.0) packages. For multiple regression, confirmatory factor analysis (Lavaan) reduced QoL measures (EQ5D5L numeric and EQ5D5L‐VAS, and LSQ‐Q1) to a single latent QoL variable (termed the QoL‐VAR).^13^ Fatigue scores (correlated at *r* = 0.91, *p* < 0.0001) were condensed to a single average z‐score. The regression model included age, time to immunotherapy and time from disease onset, excluding multicollinear variables (variance inflation factor >5). Exploratory factor analysis (EFA) reduced the battery to components capturing the highest variance.

### Study data and permissions

Informed written consent (Research Ethics Committee approval 16/YH/0013) was obtained from all patients.

## Results

In our previous report,^4^ 32% (18 out of 56) exhibited cognitive impairment as assessed by the Addenbrooke's Cognitive Examination (ACE‐III), 19% (11 out of 58) showed depression, 33% (19 out of 58) anxiety and 52% (16 out of 31) fatigue (Fatigue Scale for Motor and Cognitive Function; FSMC). Overall, 63% (38 out of 60) were impaired on at least one included domain.

**Figure 1:**
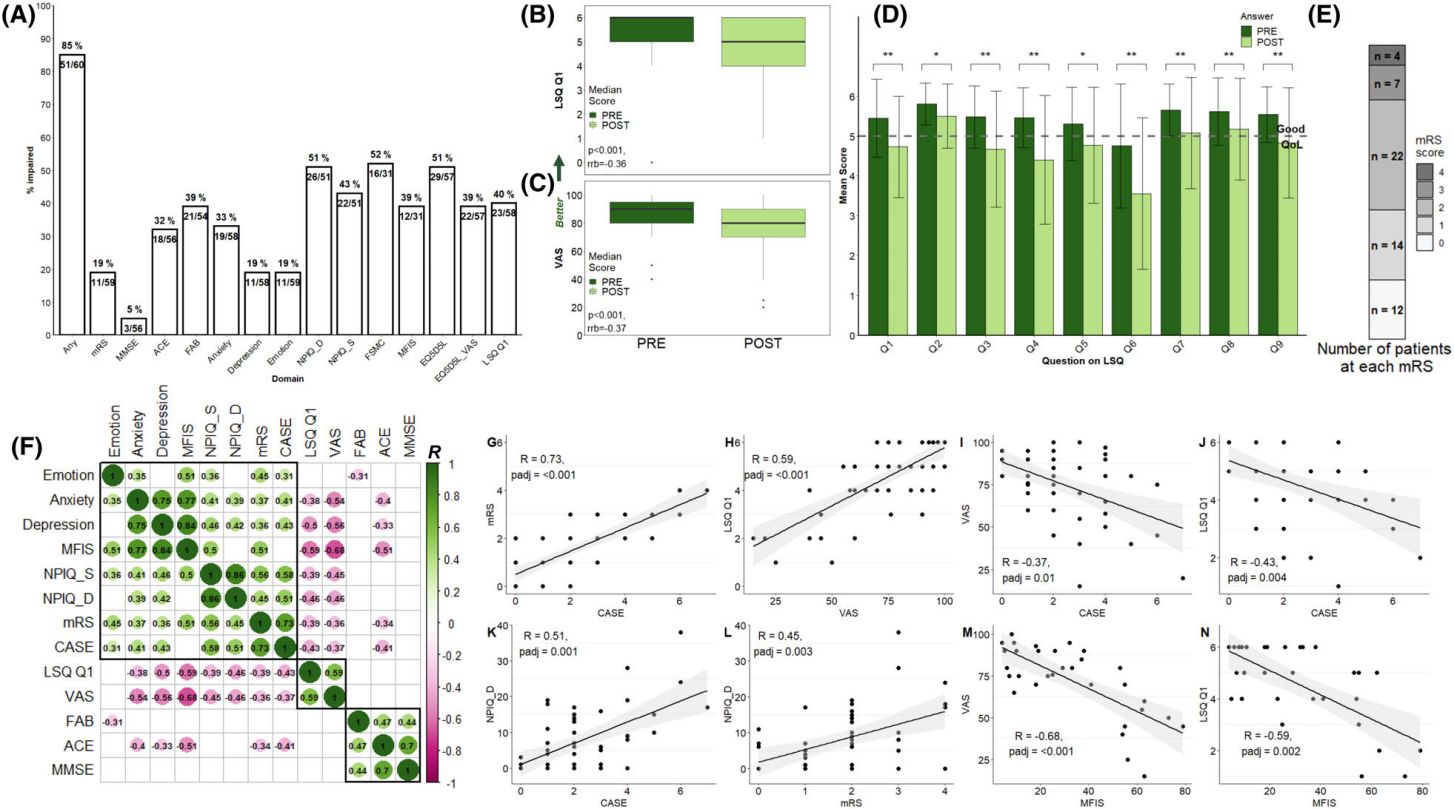
Multi‐domain impairment and decrease in quality of life after LGI1‐Ab‐E. (A) Bar chart depicting proportion of patients with abnormal scores in assessments compared to age‐appropriate cut‐offs in manuals or published literature. Labelling denotes number of patients impaired on each assessment, total number of patients having completed the assessment and % impaired. Cut‐off for anxiety and/or depression on the HADS was set at >8. (B and C): Box plots of pre‐ or peak‐ to post‐acute illness score in LGI1‐Ab‐E patients on the LSQ‐Q1 (B) and EQ5D5L‐VAS (C). The midpoint of the boxplot represents the median. Upper and lower hinges represent the 75th and 25th percentiles, respectively. Whiskers extend to the largest and smallest values within 1.5*interquartile range (IQR). Outliers beyond 1.5*IQR are depicted as circles plotted beyond the whiskers. (D) Bar chart of pre‐ to post‐acute illness score in LGI1‐antibody encephalitis patients in the nine sub‐domains of the LSQ. Q1: life as a whole; Q2: self‐care; Q3; leisure; Q4; vocation; Q5; finances; Q6; sexual life; Q7: partnership relationship; Q8: family life; Q9: contact with friends. Dotted line marks cut‐off for impairment. Error bars depict standard deviation. The higher the score, the better the outcome; a score of 5 or above is equal to a good level of satisfaction (dotted line). *p* Values calculated using Wilcoxon signed rank nonparametric test, Holm corrected. Effect size calculated by rank biserial r, rrb. *p < 0.05, **p < 0.01 (E) Stacked bar chart showing number of patients at each mRS at follow‐up; a score of 2 or below is generally considered as a good outcome. (F) Correlogram displaying relationships across all assessed domains. Positive correlations are depicted in green and negative correlations in purple. The size of the circle is proportional to the strength of the correlation with the R value depicted in each circle. Only correlations which remained significant after correction for multiple comparison (*p* < 0.05) are shown; empty boxes represent non‐significant correlations. To avoid multicollinearity, fatigue is represented by MFIS, and QoL by the LSQ‐Q1 and EQ5D5L‐VAS, in correlation plots and analysis. (G–N) Scatter plots of selected single correlations between clinician and patient‐rated outcome measures (G–J), the NPIQ‐D and clinician‐rated outcome measures (K and L) and Fatigue and QoL (M and N). ACE, Addenbrooke's Cognitive Examination; CASE, Clinical Assessment Scale in Autoimmune Encephalitis; EQ5D5L – VAS, EQ5D5L – Visual Analogue Scale; FAB, Frontal Assessment Battery; FSMC, Fatigue Scale for Motor and Cognitive Function; HADS‐A or D, Hospital Anxiety and Depression Scale – Anxiety or Depression; LGI1‐Ab‐E, leucine‐rich glioma‐inactivated 1‐antibody encephalitis; LSQ, Life Satisfaction Questionnaire; MFIS, Modified Fatigue Impact Scale; MMSE, mini‐mental state examination; mRS, modified Rankin Scale; NPIQ_D/NIPQ_S, Neuropsychiatric Inventory Questionnaire – Distress/Severity.

By adding data on emotionality, QoL and carer burden, forming a comprehensive battery of long‐term outcomes, we now observed 85% (51 out of 60) had at least one abnormal result (Fig. 1A). Within this, carer distress in spouses or relatives was common (26 out of 51; 51%), with 43% (22 out of 51) rating the patient's symptoms as severe. Excess emotionality was identified in 11 out of 59 patients (19%). QoL was reduced in 51% (29 out of 57) and significantly reduced when comparing pre‐morbid to current states (corrected *p* < 0.001 for both EQ5D5L‐VAS and LSQ‐Q1; Fig. 1B,C). Also, significant reductions in all individual QoL domains were observed (Fig. 1D and Table S4), at a time when most patients (81%, 48 out of 59) showed a functionally ‘good’ mRS of ≦2 (Fig. 1E).^4^ The mean decrease in QoL domain scores of −0.71 (range − 0.31 to −1.19; Fig. 1D and Table S4) dropped QoL below the threshold for ‘good’ in the categories of life as a whole, leisure activities, vocational life, financial situation and contact with friends.

To understand inter‐relationships between individual neurocognitive assessments and QoL, we analysed correlations after FDR correction (all significant correlations in Fig. 1F, with individual depictions in Fig. 1G–M). First, we observed close and statistically significant correlations between the two clinician‐rated assessments mRS and CASE (*R* = 0.73, *p*adj ≤0.001) and two patient‐rated outcomes, the EQ5D5L‐VAS and LSQ Q1 (*R* = 0.59, *p*adj ≤0.001; Fig. 1G,H). However, patient‐ and clinician‐rated outcomes were less strongly correlated (*R* = −0.35 to −0.43) (Fig. 1F,I,J). Multiple measures correlated with carer burden (NPIQ‐D), including mRS and CASE (Fig. 1K,L), depression and anxiety (Fig. 1F).

Emotionality was associated most strongly with fatigue (*R* = 0.51, *p*adj = 0.002;Fig. 1F). The LSQ‐Q1 and EQ5D5L‐VAS also correlated strongly with fatigue (*R* = −0.59, *p*adj = 0.002 and *R* = −0.68, *p*adj ≤0.001; Fig. 1M,N), depression (*R* = −0.5 and − 0.56, *p* ≤ 0.001), anxiety (*R* = ‐0.38 and − 0.54, *p*adj = 0.008 and <0.001) and carer burden (NPIQ‐D; both *R* = −0.46, *p* = 0.003; Fig. 1F). As previously reported, fatigue correlated with mood (*R* = 0.77, *p*adj ≤0.001 with anxiety and *R* = 0.84, *p*adj ≤0.001 with depression;Fig. 1F).

Finally, mRS and CASE correlated with multiple patient‐rated factors, whereas classical cognitive scales such as the Frontal Assessment Battery (FAB), mini‐mental state examination (MMSE) and ACE showed relatively few relationships outside of themselves.

Next, we applied hierarchical clustering to highlight over‐arching relationships. Three clusters emerged: patient‐related QoL, cognition scales and the remaining multi‐domain assessments (Fig. 1F). As patient QoL correlated with many domains outside of its immediate cluster, multiple regression, enter method, was performed to understand the most influential factors. When including covariates (age, time to immunotherapy and months since onset), the model with latest mRS and acute CASE significantly predicted QoL (F (10, 12)=5.69, *p* = 0.003, *R* ^2^ = 0.826, *R* ^2^_*adjusted*_ = 0.681) with fatigue (*p* = 0.025) and the FAB (*p* = 0.033) as the only significant factors (Table S5). Stepwise regression model confirmed the fatigue Z‐score, but not the FAB, as significant (Table S6), as did a further enter model using latest CASE and peak mRS (Table S7). There was no influence of anxiety, depression, immunotherapy or anti‐seizure medications on fatigue (Table S3).

Finally, as the research battery required more than 3 hours, its core components were distilled. Using EFA, most QoL‐VAR variance was captured by five instruments: the ACE, HADS, CASE, Modified Fatigue Impact Scale (MFIS) and EQ5D5L‐VAS (Table S8). Cumulatively, these took ~30 min to complete. Subsequent regression analysis showed the ACE, HADS, CASE and MFIS predicted 66% of EQ5DL‐VAS variance (F(4,24) = 11.63, *p* = 0.0001, *R* ^2^ = 0.660, *R* ^2^_*adjusted*_ = 0.603), showing these real‐life instruments accurately reflected the QoL‐VAR variable.

## Discussion

To our knowledge, this study represents the most detailed exploration of cognitive, neuropsychiatric and QoL outcomes, and the first systematic survey of carer distress, in LGI1‐Ab‐E. Our data expand the proportion of patients impacted from 63% to 85%.^4^ Moreover, they highlight the substantial burden placed on carers and spouses; >50% described psychological distress, warranting increased societal acknowledgement, practical and emotional support and improved attention to neurorehabilitation in AE. Correlations, corrected for multiple comparisons, revealed interactions between QoL and mood, fatigue, emotionality and carer burden, but not more traditionally investigated cognitive outcomes.

We uncovered a marked pre‐ to post‐LGI1‐Ab‐E decline in QoL, including all aspects of contentment quantified by the LSQ, despite satisfactory clinical outcomes such as a ‘good’ mRS (mean post‐illness score = 1.6). These results reinforce that global measures of functional disability such as the mRS are inadequate in LGI1‐Ab‐E.

Fatigue was significant in all our regression models and the principal and only consistent QoL predictor, independent of depression. Time to immunotherapy did not influence QoL, despite its efficacy in acute seizure control.^3^ Fatigue is recognised as a disabling feature of many long‐term neurological conditions.^14^ Our results signal the importance of recognising fatigue in LGI1‐Ab‐E and a multidisciplinary team approach. Further studies will be required to understand its pathogenesis and treatment, including the possibility of randomised controlled trials of fatigue management post‐AE.

Finally, our data identify a shortened, effective battery comprising five assessments with future utility in both clinic and trial settings.

### Strengths and limitations

Strengths include study size and strict phenotyping, and the breadth and depth of hypothesis‐driven assessments drawn from our own clinical observations, the AE literature^5^, ^6^, ^7^ and other neuro‐inflammatory diseases.^15^ Potential drawbacks include the UK‐only cohort, heterogeneity in follow‐up durations, pragmatic retrospective ascertainment of pre‐illness status, lack of sleep assessments and an important limitation that fatigue questionnaires were only administered to a subset (31 out of 60, 52%) of patients. This subset had a significantly shorter mean illness duration by almost three years, but this was accounted for in the regression model, and there were no other significant differences between the groups with and without fatigue data, including comparable current mRS and CASE scores (Table S3). While 85% of patients demonstrated at least one abnormal score in these assessments, around 2.5% of the general population are also likely to show abnormalities, especially across our wide‐ranging assessment protocol. The brief battery presented here is verified only in patients with LGI1‐Ab‐E, and future work should evaluate its efficacy in patients with other forms of antibody‐mediated encephalitis.

## Conclusions

Our findings provide the first detailed examination of QoL in LGI1‐Ab‐E and should cement the importance of assessing QoL in AE. Our findings demonstrate fatigue is the major driver of reduced QoL Further, our simple multimodal battery should assist a more accurate capture of patient outcomes and help standardise international research.

## Author Contributions

The corresponding author (Professor Sarosh R. Irani) had full access to all the data in the study and takes responsibility for the integrity of the data and the accuracy of the data analysis. Data analysis was conducted by Dr Veldsman, Dr Binks, Professor Irani and Professor Husain. Professor Irani confirms that all authors meet the ICMJE criteria for authorship.

## Acknowledgements

None.

## Conflict of Interest

SRI is a co‐applicant and receives royalties on patent application WO/2010/046716 (U.K. patent no. PCT/GB2009/051441) entitled ‘Neurological Autoimmune Disorders’. The patent has been licenced commercially for the development of assays for LGI1 and other VGKC‐complex antibodies. SRI and SB are co‐applicants on a patent application entitled ‘Diagnostic Strategy to improve specificity of CASPR2 antibody detection’ (PCT/GB2019/051257, publication number WO/2019/211633 and UK1807410.4). SRI has received honoraria from UCB, MedImmun, ADC therapeutics and Medlink Neurology, and research support from CSL Behring, UCB and ONO Pharma. AEH has received research support from UCB Pharma. JC has received speaking honoraria from Bial, Brittania, advisory board fees from Bial and Teva and is on a patent for a test of GABA_A_ antibodies. SJ has served as an international advisory board member for Alexion, Alnylam, Argenx, Regeneron, Immunovant and UCB pharmaceuticals, is currently an expert panel member of Myasthenia Gravis consortium for Argenx pharmaceuticals and has received speaker fees from Argenx, Eisai, Terumo BCT and UCB pharmaceuticals. SR serves as a consultant on an advisory board for UCB and Limbic Neurology, and has been an invited speaker for Biogen, Alexion, Novartis, Excemed and Limbic Neurology. AE has received no personal honoraria, but The Encephalitis Society has received sponsorship/honoraria/speaker fees from Valneva, Roche, UCB, Pfizer, Biomerieux, CSL Behring, Svar and EuroImmun. MIL has no conflicts of interest relevant to encephalitis. MB has received research support from Aptinyx Inc and honoraria from Medlink Neurology. MH has received fees for a lecture delivered to Lilly UK and for consultancy to Otsuka Pharmaceuticals. MV, PM, SM, MSN and DO have no conflicts of interest to declare.

## Funding Information

Professor Irani is supported by the Wellcome Trust (104079/Z/14/Z), BMA Research Grants – Vera Down grant (2013), Margaret Temple (2017), Epilepsy Research UK (P1201), the Fulbright UK‐US commission (MS Society research award) and by the NIHR Oxford Biomedical Research Centre. Dr Binks is currently supported by the NIHR through a Clinical Lectureship and has received support from the Wellcome Trust. Dr Veldsman is currently an employee of Cambridge Cognition. Dr Handel is supported by the MRC (MR/X022013/1) and Oxford Health BRC. Associate Professor Sudarshini Ramanathan has received research funding from the National Health and Medical Research Council (NHMRC, Australia), the Petre Foundation, the Brain Foundation (Australia), the Royal Australasian College of Physicians and the University of Sydney. She is currently supported by an NHMRC Investigator Grant (GNT2008339). Professor Husain is supported by the Wellcome Trust (206330/Z/17/Z) and by the NIHR Oxford Biomedical Research Centre. The views expressed are those of the author(s) and not necessarily those of the NHS, the NIHR or the Department of Health. The funders had no role in the design and conduct of the study; collection, management, analysis and interpretation of the data; preparation, review or approval of the manuscript; and decision to submit the manuscript for publication.

## Supporting information


Data S1.
