# Supplementary material for: Fatigue predicts quality of life after leucine‐rich glioma‐inactivated 1‐antibody encephalitis
Source: Ann Clin Transl Neurol. 2024 Feb 1;11(4):1053–8. doi: 10.1002/acn3.52006 (PMC11021603; doi:10.1002/acn3.52006)
Supplement: Supplementary file 1 — Data S1. [file ACN3-11-1053-s001.docx]

**Supplementary Table 1:** Baseline demographics of 60 patients with leucine-rich glioma-inactivated 1 antibody encephalitis (LGI1-Ab-E)

| **Demographics** | **Median/mean (range, IQR)** | **Number of patients (%)** |
| --- | --- | --- |
| **Median age at onset (range, IQR)** | 64 (44-86, 15.25) | 60 (n/a) |
| **Median age at assessment (range, IQR)** | 70 (44-92, 18.75) | 60 (n/a) |
| **Assessment months post onset (range, IQR)** | 41 (4-179, 73) | 60 (n/a) |
| **Female** | - | 20/60 (33%) |
| **Functional status** |  |  |
| **Current mRS, mean (range)** | 1.6 (0-4) | 59 |
| **mRS >2** | - | 11/59 (19) |
| **Abbreviations:** IQR, interquartile range; mRS, modified Rankin Scale | | |

**Supplementary Table 2:** Full list of assessments administered in this and previous study (1)

| **Assessment** | **Domain assessed** |
| --- | --- |
| Addenbrooke’s Cognitive Examination(2) | Cognition |
| Mini-mental State Examination(3) | Cognition |
| Frontal Assessment Battery(4) | Cognition |
| Pathological Laughter and Crying Scale(5) | Emotionality |
| Hospital Anxiety and Depression Scale(6) | Affective symptoms |
| Modified Rankin Scale(7) | Clinician-rated disability |
| Clinical Assessment Scale in Autoimmune Encephalitis(8) | Clinician-rated disability |
| EQ5D5L(9) | Quality of Life |
| Life Satisfaction Questionnaire(10) | Quality of Life |
| Fatigue Scale for Motor and Cognitive Function(11) | Fatigue |
| Modified Fatigue Impact Scale(12) | Fatigue |
| Neuropsychiatric Inventory Questionnaire(13,14) | Carer-rating & distress |

**References:**

1. Binks SNM, Veldsman M, Easton A, Leite MI, Okai D, Husain M, et al. Residual Fatigue and Cognitive Deficits in Patients after Leucine-Rich Glioma-Inactivated 1 Antibody Encephalitis. JAMA Neurol. 2021;78(5):617–9.

2. Mioshi E, Dawson K, Mitchell J, Arnold R, Hodges JR. The Addenbrooke’s Cognitive Examination revised (ACE-R): A brief cognitive test battery for dementia screening. Int J Geriatr Psychiatry. 2006;21(11):1078–85.

3. Folstein MF, Folstein SE, McHugh PR. “Mini-mental state”. A practical method for grading the cognitive state of patients for the clinician. J Psychiatr Res [Internet]. 1975 Nov;12(3):189–98.

4. Dubois B, Slachevsky A, Litvan I, Pillon B. The FAB: A frontal assessment battery at bedside. Neurology. 2000 Dec 12;55(11):1621–6.

5. Robinson G, Parikh M, Lipsey JR, Starkstein SE, Price R. Pathological laughing and crying following stroke: validation of a measurement scale and a double-blind treatment study. Am J Psychiatry. 1993 Feb;150(2):286–93.

6. Zigmond AS, Snaith RP. The Hospital Anxiety and Depression Scale. Acta Psychiatr Scand. 1983 Jun;67(6):361–70.

7. Rankin J. Cerebral Vascular Accidents in Patients over the Age of 60: II. Prognosis. Scott Med J. 1957 May 25;2(5):200–15.

8. Lim JA, Lee ST, Moon J, Jun JS, Kim TJ, Shin YW, et al. Development of the clinical assessment scale in autoimmune encephalitis. Ann Neurol. 2019;85(3):352–8.

9. Szende A, Janssen B, Cabasés J. Self-reported population health: An international perspective based on EQ-5D. Self-Reported Population Health: An International Perspective Based on EQ-5D. 2014. 1–196 p.

10. Fugl-Meyer AR, Bränholm IB, Fugl-Meyer KS. Happiness and domain-specific life satisfaction in adult northern Swedes. Clin Rehabil. 1991;5(1):25–33.

11. Penner IK, Raselli C, Stöcklin M, Opwis K, Kappos L, Calabrese P. The Fatigue Scale for Motor and Cognitive Functions (FSMC): Validation of a new instrument to assess multiple sclerosis-related fatigue. Mult Scler. 2009;15(12):1509–17.

12. Fisk JD, Ritvo PG, Ross L, Haase DA, Marrie TJ, Schlech WF, et al. Measuring the Functional Impact of Fatigue : Initial Validation of the Fatigue Impact Scale. Clin Infect Dis. 1994;18(1):S79–83.

13. Kaufer DI, Cummings JL, Ketchel P, Smith V, MacMillan A, Shelley T, et al. Validation of the NPI-Q, a brief clinical form of the Neuropsychiatric Inventory. J Neuropsychiatry Clin Neurosci. 2000;12(2):233–9.

14. Leonard M, McInerney S, McFarland J, Condon C, Awan F, O’Connor M, et al. Comparison of cognitive and neuropsychiatric profiles in hospitalised elderly medical patients with delirium, dementia and comorbid delirium-dementia. BMJ Open. 2016;6(3):1–9.

**Supplementary Methods – supplementary statistical methods**

Pre- and current post-acute illness scores were compared retrospectively for overall outcome measures (EQ5D5L-VAS, LSQ question 1, mRS and CASE) using t-tests (for normally distributed scores) and Wilcoxon signed-rank t-tests (for non-parametric scores), reporting the Rank-Biserial (r_rb_) correlation as a measure of effect size. Statistics were conducted in JASP (2019; Version 0.10.2) and/or R (v4.0.3).

Correlations (Spearman method) and clustering were performed and plotted in R (v4.0.3) with hmisc (v5.1.1), corrplot (v0.92) and ggpubr (v0.6.0) packages. Pairwise complete observations were used as it was already established that there was no difference in the groups with and without fatigue questionnaires, other than a shorter time since illness duration. False discovery rate (FDR) was used to correct for multiple comparisons. Hierarchical clustering was performed in corrplot, based on three factors as identified by the Exploratory Factor Analysis.

Given the multiple collected measures of QoL, confirmatory factor analysis in Lavaan reduced these to a single latent QoL variable (QoL-VAR). A threshold of root mean square error of approximation (RMSEA) <0.05 was employed to assess model fit. The two fatigue scores were condensed to a single measure, and the average Z-score of the total score for each scale was used.

Multiple regression, using the enter method, was conducted in JASP. A variance inflation factor threshold of greater than five was employed to guide which variables to exclude on the basis of multicollinearity. The multiple regression included age, time to immunotherapy and time from disease onset to ensure the model controlled for these potential confounding variables. To minimise collinearity of variables, the medication effect was examined separately by ANOVA. Also, stepwise regression was run to ensure our model was not impacted by the variables removed due to multicollinearity in the enter method model. In the stepwise regression, variables were sequentially removed based on meeting a significance threshold (p<0.05).

Exploratory factor analysis was used to reduce the test battery to only the tests capturing the highest variance. Varimax orthogonal rotation and parallel analysis were employed to determine the number of factors to retain. Tests were chosen with the highest loadings per factor to include in a reduced battery. Multiple regression, enter method, was used to estimate the proportion of variance in the key outcome measure, QoL-VAR, captured by the reduced battery.

**References**

Hair, JF Jr, Anderson RE, Tatham RL, Black WC. Multivariate Data Analysis. 3rd ed. New York: Macmillan; 1995.

Rosseel Y. lavaan: An R Package for Structural Equation Modeling. J Stat Softw. 2012 May 24;48(1):1–36.

Wei T, Simko V. An introduction to corrplot Package (on-line). 2021 Nov 18 (accessed 2023 Oct 6): Available from: <https://cran.r-project.org/web/packages/corrplot/vignettes/corrplot-intro.html>

**Supplementary Table 3. Differences between patients with and without fatigue measures.^a^**

| **Factor** | **Mean – without fatigue (n=29)^b^** | **Mean – with fatigue (n=31)^b^** | **Statistic^c^** | **df** | **p** | **Cohen’s d^d^** |
| --- | --- | --- | --- | --- | --- | --- |
| Age at onset | 65.03 (29) | 63.65 (31) | 0.489 | 57.13 | 0.627 | 0.126 |
| Age at visit | 71.31 (29) | 66.55 (31) | 1.643 | 57.99 | 0.106 | 0.424 |
| Months since onset | 75.41 (29) | 37.74 (31) | 3.270 | 51.74 | 0.002^e^ | 0.849 |
| Peak mRS | 3.10 (29) | 3.10 (30) | 0.012 | 50.57 | 0.991 | 0.003 |
| Time to IT / weeks | 31.08 (24) | 19.14 (28) | 1.134 | 27.74 | 0.267 | 0.325 |
| Acute CASE score | 5.75 (28) | 6.76 (29) | -1.112 | 53.62 | 0.271 | -0.294 |
| LSQ-Q1-post | 4.7 (27) | 4.48 (31) | 0.611 | 54.08 | 0.544 | 0.159 |
| EQ5D5L-VAS-post | 78.52 (27) | 72.39 (31) | 1.150 | 55.96 | 0.255 | 0.301 |
| HADS total score | 8.48 (27) | 11 (31) | -1.246 | 55.66 | 0.218 | -0.326 |
| ACE | 88.65 (26) | 88.97 (30) | -0.150 | 53.66 | 0.881 | -0.040 |
| MMSE | 28.31 (26) | 28.23 (30) | 0.126 | 53.02 | 0.900 | 0.034 |
| FAB | 15.08 (25) | 15.97 (29) | -1.573 | 44.76 | 0.123 | -0.433 |
| NPIQ severity | 5.5 (24) | 7.78 (27) | -1.324 | 44.88 | 0.192 | -0.367 |
| Emotionality | 5.5 (28) | 7.26 (31) | -0.963 | 54.01 | 0.340 | -0.249 |
| QoL latent variable | 0.135 (27) | -0.118 (31) | 0.963 | 55.98 | 0.340 | 0.252 |
| Latest mRS | 1.6 (29) | 1.7 (30) | -0.381 | 53.85 | 0.704 | -0.0995 |
| Latest CASE | 1.89 (28) | 2.34 (29) | -1.015 | 54.75 | 0.315 | -0.269 |
|  | **Yes/no (n=29)^g^** | **Yes/no (n=31)^g^** |  |  |  |  |
| Presence of focal seizures (yes/no)^f^ | 17/12 (29) | 22/8 (30) | 1.424 | 1 | 0.233 | -0.29 |

^a^Independent Samples T-Tests, except for presence of focal seizures – Chi Square test.^b^For each column numbers show: mean (total number with data).^c^T-value or Chi Squared. ^d^measure of effect size. ^e^Significant p-value. ^f^Presence of focal seizures (not including generalised seizures or faciobrachial dystonic seizures).^g^For each column numbers show numbers with focal seizures/without focal seizures (total with data**)**. **Note:** A potential confounding effect of medication was examined. However, there was neither a difference in fatigue (estimated by ANOVA; F(4,25)=0.78, p=0.551) if patients had received none, one, two or three immunotherapies (steroids, intravenous immunoglobulins or plasma exchange), nor between fatigue and number of anti-epileptic drugs (AEDs; r=0.08, p=0.675). Specifically, patients taking valproate and/or levetiracetam showed no significant increase in fatigue compared to patients taking other AEDs or none (independent t-test, t(28)=-1.692, p=0.102). Similarly, psychotropic drugs did not raise the likelihood of scoring worse on fatigue measures compared to others in the cohort (p=0.128). However, the medicated group showed higher HADS depression scores and lower QoL (p= 0.004 and 0.005, respectively; independent samples T-test corrected for multiple comparisons).

**Abbreviations:** mRS, modified Rankin Scale; IT, immunotherapy; CASE, Clinical Assessment Score in Autoimmune Encephalitis; LSQ-Q1, Life Satisfaction Questionnaire Question 1; EQ5D5L-VAS, EQ5D5L Visual Analogue Scale; HADS, Hospital Anxiety and Depression Scale; ACE, Addenbrooke’s Cognitive Examination; MMSE, Mini Mental State Examination; FAB, Frontal Assessment Battery; NPIQ, Neuropsychiatric Inventory Questionnaire; QoL, Quality of Life; df, degrees of freedom

**Supplementary Table 4. Changes in LSQ domains pre- and post LGI1-Ab-E.**

| **Question** | **Mean score pre** | **Mean score post** | **Change** | **p-val adj*** |
| --- | --- | --- | --- | --- |
| Q1: Life as a whole | 5.44 | 4.73 | -0.71 | 0.002 |
| Q2: Self care | 5.81 | 5.5 | -0.31 | 0.04 |
| Q3: Leisure | 5.48 | 4.67 | -0.81 | 0.001 |
| Q4: Vocation | 5.46 | 4.40 | -1.06 | 0.0007 |
| Q5: Finances | 5.31 | 4.77 | -0.54 | 0.04 |
| Q6: Sexual life | 4.75 | 3.56 | -1.19 | 0.0006 |
| Q7: Partnership relationship | 5.65 | 5.08 | -0.57 | 0.006 |
| Q8: Family life | 5.62 | 5.17 | -0.45 | 0.01 |
| Q9: Contact with friends | 5.54 | 4,83 | -0.71 | 0.001 |
| Mean change | NA | NA | -0.71 | NA |

*paired Wilcoxon test performed on 52 patients with pre- and post- test results for all domains, Holm adjusted for multiple comparisons

**Supplementary Table 5: Full multiple regression analysis of factors which predict Quality of Life with the QoL latent variable** **(QoL-VAR)** **as the dependent variable.^a^** Fatigue remained a significant factor upon substituting the LSQ-Q1 (F(10,12)=2.814 p=0.047, R^2^=0.701, R^2^*_adjusted_*=0.452) and the EQ5D5L-VAS (F(10,12)=4.802, p=0.006, R^2^=0.800, R^2^*_adjusted_*=0.633) for the QoL-VAR, providing orthogonal validation of the QoL-VAR.

| **Model summary** | | | | | | | |
| --- | --- | --- | --- | --- | --- | --- | --- |
| **Model** |  |  |  |  |  |  |  |
| **1** | **R** | **R^2^** | **Adjusted R^2^** | **RMSE** |  |  |  |
|  | 0.909 | 0.826 | 0.681 | 0.581 |  |  |  |
| **ANOVA** | | | | | | | |
| **Model** |  | **Sum of Squares** | **df** | **Mean Square** | **F** | **p** |  |
| **1** | **Regression** | 19.227 | 10 | 1.923 | 5.694 | 0.003^b^ |  |
|  | **Residual** | 4.052 | 12 | 0.338 |  |  |  |
|  | **Total** | 23.279 | 22 |  |  |  |  |
|  |  |  |  |  |  |  |  |
| **Co-efficients** |  |  |  |  |  |  | **Collinearity statistic** |
| **Model** |  | **Unstandardised** | **Standard Error** | **Standardised** | **t** | **p** | **VIF** |
| **1** | **(Intercept)** | 6.863 | 4.216 |  | 1.628 | 0.130 |  |
|  | **mRS – now** | -0.213 | 0.226 | -0.194 | -0.943 | 0.364 | 2.908 |
|  | **Age at visit** | 0.004 | 0.014 | 0.046 | 0.315 | 0.758 | 1.486 |
|  | **Time to IT / weeks** | 0.004 | 0.010 | 0.071 | 0.414 | 0.686 | 2.032 |
|  | **CASE score – acute** | -0.053 | 0.034 | -0.206 | -1.565 | 0.143 | 1.199 |
|  | **NPIQ severity** | -0.032 | 0.026 | -0.239 | -1.235 | 0.241 | 2.583 |
|  | **Fatigue Z-score** | -0.668 | 0.261 | -0.637 | -2.563 | 0.025^b^ | 4.262 |
|  | **ACE** | -0.012 | 0.036 | -0.073 | -0.321 | 0.754 | 3.615 |
|  | **HADS** | -0.021 | 0.034 | -0.172 | -0.623 | 0.545 | 5.246 |
|  | **Months since onset** | -0.008 | 0.005 | -0.292 | -1.567 | 0.143 | 2.399 |
|  | **FAB** | -0.304 | 0.126 | -0.559 | -2.406 | 0.033^b^ | 3.719 |

Abbreviations: QoL, Quality of Life; RMSE, root mean square error; df, degrees of freedom; VIF, variance inflation factor; mRS, Modified Rankin Scale; IT, immunotherapy; IT, immunotherapy; CASE, Clinical Assessment Scale in Autoimmune Encephalitis; NPIQ, Neuropsychiatric Inventory Questionnaire; ACE = Addenbrooke’s Cognitive Examination; HADS, Hospital Anxiety and Depression Scale; FAB, Frontal Assessment Battery.

^a^ Multiple regression, enter model. ^b^Statistically significant p values.

**Supplementary Table 6: Stepwise regression predictive of QoL in LGI1-Ab-E with the QoL latent variable** **(QoL-VAR)** **as the dependent variable.**

| **Model summary** | | | | | | | |
| --- | --- | --- | --- | --- | --- | --- | --- |
| **Model** |  |  |  |  |  |  |  |
|  | **R** | **R^2^** | **Adjusted R^2^** | **RMSE** |  |  |  |
| **1** | 0.000 | 0.000 | 0.000 | 0.928 |  |  |  |
| **2** | 0.796 | 0.634 | 0.617 | 0.575 |  |  |  |
|  | | | | | | | |
| **ANOVA**^a^ | | | | | | | |
| **Model** |  | **Sum of Squares** | **df** | **Mean Square** | **F** | **p** |  |
| **1** | **Regression** | 12.023 | 1 | 12.023 | 36.387 | <0.001^b^ |  |
|  | **Residual** | 6.939 | 21 | 0.330 |  |  |  |
|  | **Total** | 18.962 | 22 |  |  |  |  |
|  |  |  |  |  |  |  |  |
| **Co-efficients** |  |  |  |  |  |  |  |
| **Model** |  | **Unstandardised** | **Standard Error** | **Standardised** | **t** | **p** |  |
| **1** | **(Intercept)** | -0.028 | 0.194 |  | -0.142 | 0.888 |  |
| **2** | **(Intercept)** | 2.713 | 0.470 |  | 5.774 | <0.001^b^ |  |
|  | **Fatigue** | -0.754 | 0.125 | -0.796 | -6.032 | <0.001^b^ |  |

Abbreviations: RMSE, root mean square error; df, degrees of freedom

^a^The intercept model is omitted, as no meaningful information can be shown ^b^Statistically significant p values.

Covariates considered but not included: mRS, Age at Visit, Time to IT/weeks, CASE score acute, NPIQ Severity, ACE, HADS, Months since onset, FAB

**Supplementary Table 7: Full multiple regression analysis^a^ of factors which predict Quality of Life with the QoL latent variable** **(QoL-VAR)** **as the dependent variable, substituting peak mRS and acute CASE for mRS now and CASE now**

| **Model summary** | | | | | | | |
| --- | --- | --- | --- | --- | --- | --- | --- |
| **Model** |  |  |  |  |  |  |  |
|  | **R** | **R^2^** | **Adjusted R^2^** | **RMSE** |  |  |  |
| **H1** | 0.903 | 0.815 | 0.661 | 0.541 |  |  |  |
| **ANOVA** | | | | | | | |
| **Model** |  | **Sum of Squares** | **df** | **Mean Square** | **F** | **p** |  |
| **1** | **Regression** | 15.453 | 10 | 1.545 | 5.284 | 0.004^b^ |  |
|  | **Residual** | 3.509 | 12 | 0.292 |  |  |  |
|  | **Total** | 18.692 | 22 |  |  |  |  |
|  |  |  |  |  |  |  |  |
| **Co-efficients** |  |  |  |  |  |  | **Collinearity statistic** |
| **Model** |  | **Unstandardised** | **Standard Error** | **Standardised** | **t** | **p** | **VIF** |
| **H1** | **(Intercept)** | 7.083 | 3.789 |  | 1.869 | 0.086 |  |
|  | **mRS – peak** | -0.037 | 0.143 | -0.039 | -0.259 | 0.800 | 1.444 |
|  | **Age at visit** | 0.006 | 0.015 | 0.066 | 0.386 | 0.706 | 1.868 |
|  | **Time to IT / weeks** | 0.002 | 0.005 | 0.069 | 0.441 | 0.667 | 1.587 |
|  | **CASE score - post** | -0.128 | 0.104 | -0.215 | -1.229 | 0.243 | 1.977 |
|  | **NPIQ severity** | -0.015 | 0.023 | -0.126 | -0.653 | 0.526 | 2.411 |
|  | **Fatigue Z-score** | -0.687 | 0.247 | -0.726 | -2.783 | 0.017^b^ | 4.410 |
|  | **ACE** | -0.010 | 0.038 | -0.071 | -0.269 | 0.792 | 4.546 |
|  | **HADS** | -0.005 | 0.033 | -0.041 | -0.140 | 0.891 | 5.485 |
|  | **Months since onset** | -0.006 | 0.003 | -0.262 | -1.768 | 0.102 | 1.425 |
|  | **FAB** | -0.207 | 0.108 | -0.422 | -1.923 | 0.078 | 3.121 |

Abbreviations: QoL, Quality of Life; RMSE, root mean square error; df, degrees of freedom; VIF, variance inflation factor; mRS, Modified Rankin Scale; IT, immunotherapy; IT, immunotherapy; CASE, Clinical Assessment Scale in Autoimmune Encephalitis; NPIQ, Neuropsychiatric Inventory Questionnaire; ACE = Addenbrooke’s Cognitive Examination; HADS, Hospital Anxiety and Depression Scale; FAB, Frontal Assessment Battery.

^a^ Multiple regression, enter model. ^b^Statistically significant p values.

**Supplementary Table 8. Exploratory factor analysis of the assessment battery.** In bold are the highest loadings for each factor chosen for the reduced battery. Empty cells within a factor column show that the assessment was not ascribed to that factor. Varimax orthogonal rotation applied, number of factors determined by parallel analysis. Abbreviations: MFIS, Modified Fatigue Impact Scale; EQ5D5L-VAS, EQ5D5L visual analogue scale; HADS, Hospital Anxiety and Depression Scale; CASE, Clinical Assessment Scale for Autoimmune Encephalitis; ACE, Addenbrooke’s Cognitive Examination; FSMC, Fatigue Scale for Motor and Cognitive Function; LSQ-Q1, Life Satisfaction Questionnaire – question 1; NPIQ, Neuropsychiatric Inventory Questionnaire; mRS, Modified Rankin Scale; MMSE, Mini-Mental State Examination; FAB, Frontal Assessment Battery.

|  | **Factor 1** | **Factor 2** | **Factor 3** |
| --- | --- | --- | --- |
| MFIS | **0.910** |  |  |
| EQ5D5L-VAS | **-0.832** |  |  |
| HADS | **0.779** |  |  |
| CASE |  | **0.880** |  |
| ACE |  |  | **0.919** |
| FSMC | 0.899 |  |  |
| LSQ-Q1 | -0.717 |  |  |
| Emotionality | 0.401 |  |  |
| NPIQ Severity |  | 0.567 |  |
| mRS |  | 0.737 |  |
| MMSE |  |  | 0.702 |
| FAB |  |  | 0.582 |
